# Successful Development and Implementation of a Large Virtual Interprofessional Education Activity Applying the Social Determinants of Health

**DOI:** 10.3390/pharmacy10060157

**Published:** 2022-11-23

**Authors:** Karl R. Kodweis, Elizabeth A. Hall, Chelsea P. Renfro, Neena Thomas-Gosain, Robin Lennon-Dearing, Jonathon K. Walker, Tyler M. Kiles

**Affiliations:** 1 Department of Clinical Pharmacy and Translational Science, College of Pharmacy, The University of Tennessee Health Science Center, Memphis, TN 38163, USA; 2 Vanderbilt Specialty Pharmacy Health Outcomes and Research Program, Vanderbilt University Medical Center, Nashville, TN 37232, USA; 3 Department of Medical Education, College of Medicine, The University of Tennessee Health Science Center, Memphis, TN 38163, USA; 4 School of Social Work, University of Memphis, Memphis, TN 38152, USA

**Keywords:** interprofessional education, technology, remote learning, social determinants of health

## Abstract

Interprofessional education (IPE) activities provide students insight into healthcare teams, shared decision-making, and social determinants of health (SDOH). Virtual IPE activities with large student populations or across multicampus have not been evaluated. The study aimed to explore the interprofessional competency growth in students, across several disciplines, following participation in a large-scale, virtual IPE activity. Students from pharmacy, medicine, social work, and physician assistant programs across Tennessee participated in an IPE patient case and SDOH in fall 2020 and fall 2021. Pre- and postsurveys included Likert ranking of 16 statements based on the 2011 Interprofessional Education Collaborative (IPEC) framework. A total of 607 students completed surveys (overall response rate, 76%). Wilcoxon signed-rank tests were performed on the pre-/postsurvey data, in aggregate and by discipline. Significant increases in all IPEC competency statements were seen, both in aggregate (100% of statements with *p* < 0.001) and in pharmacy (100% of statements with *p* < 0.001) and medicine subgroups (94% of statements with *p* < 0.001). Implementing large virtual IPE activities involving a complex patient case and SDOH significantly increased student IPEC competency outcomes for participating students, whether in aggregate or on a discipline-specific basis.

## 1. Introduction

Interdisciplinary collaboration is an essential aspect of effective patient-centered care [1,2]. Interprofessional education (IPE), where individuals from multiple professions, such as medicine, nursing, pharmacy, and social work, learn about, from, and with each other, is useful for preparing healthcare practitioners to work within a team and promotes trust-building and engagement in shared problem-solving [1,2]. Early exposure to IPE gives students a clear context of what it means to work on a healthcare team and builds confidence in their shared decision-making. IPE can also be leveraged to educate future healthcare professionals about the social determinants of health (SDOH) [3]. The conditions in which people are born, live, learn, work, worship, and age, can affect a wide range of health outcomes and risks; therefore, addressing the SDOH is a major goal of Healthy People 2030 [4]. While an understanding of the SDOH is important for each profession within their own context, designing appropriate interventions to address a patient’s social needs requires an interdisciplinary approach. Through exploring how social factors impact all aspects of care, healthcare professions students can better understand the circumstances of the patients they serve and the roles of all members of the healthcare team in addressing barriers to patient care.

Developing effective IPE activities is often resource-intensive, and conventional obstacles to implementing IPE activities include scheduling, space, faculty facilitation, and traditional professional boundaries [5,6]. However, successful virtual IPE activities have been documented that may alleviate these constraints [5,7,8,9,10]. For example, Shoemaker and colleagues have extensively investigated the use of computer-based simulated patients to teach small groups of physician assistant, physical therapy, and occupational therapy students [5,7,8]. Using an Internet-based virtual patient software, these studies found that the students experienced an understanding of the benefits of collaborative care, as well as clarification of their roles through the virtual simulation [7]. Robertson and colleagues found that an IPE activity using synchronous video conferencing was able to meet competencies for medical and nursing students, but also simplified logistics and allowed the activity to occur despite restrictions in face-to-face instruction caused by the COVID-19 pandemic [9]. Another study by Leadbeater and colleagues investigated an online interactive IPE activity among biomedical science students and medical students which also met the desired outcomes of the interprofessional experience despite being delivered completely online [10]. While these promising strategies to overcome logistic concerns have been studied, virtual IPE activities incorporating large numbers of students or multicampus educational programs have not been thoroughly investigated. Furthermore, there is a dearth of literature exploring the SDOH within the IPE context.

In response to the COVID-19 pandemic, investigators at a large Doctor of Pharmacy (PharmD) program were challenged to deliver interprofessional education via a virtual format, and there was a unique opportunity to investigate this gap. Innovative online and hybrid educational strategies have demonstrated it is possible to deliver meaningful IPE during the pandemic [9,11,12,13,14]. Given the multicampus structure of the institution and the high number of participating students, the virtual approach of this intervention helped overcome barriers previously associated with IPE activities, including geographic constraints, scheduling issues, and classroom size. Before this intervention, it would have been logistically impossible to accommodate over 400 students from four different programs and three different cities at one time in any single location, which would have necessitated scheduling and delivering this activity over multiple days.

This project describes the development and implementation of a large synchronous online case-based IPE event focused on the SDOH. The primary aim was to explore whether students from various healthcare disciplines gained interprofessional competency after participating in the activity involving the care of a complex patient. Secondarily, given that slightly different logistical approaches were used in the virtual setting during the two years of this activity, this study also aimed to determine any differences in interprofessional competency between the two years.

## 2. Materials and Methods

### 2.1. Virtual IPE Activity Development and Implementation

The patient case was previously designed as an in-person IPE activity for pharmacy, medicine, physician assistant, and social work students. The original case was developed over the course of a semester with feedback and input from faculty stakeholders. Due to the pandemic, the case was transformed into an unfolding clinical scenario and built into an interactive online format using QuestionPro and the content was further amended with stakeholder input. The virtual IPE was aligned with curricular insight from faculty and was scheduled for a two-hour time block via Zoom, three months in advance.

This virtual IPE activity focused on the team-based care of a patient, Mr. Brown with heart failure who also has many social needs during transitions of care. The inpatient team was convened to assess the patient’s unmet needs and formulate a discharge plan. Review of his records revealed six admissions in the past six months. Each time he was admitted he rapidly improved, and the team wondered why the patient required so many hospitalizations. The complex case was broken into three parts: interdisciplinary rounding in the hospital, discharge counseling, and a home visit with follow-up care. During the activity, each profession was provided with discipline-specific documents about the patient’s medical and/or social history, and the students worked together to navigate a series of questions and activities via QuestionPro. The participants worked collaboratively to fill in the answers to the ten questions in the online survey and had to answer them appropriately to access the next question. For example, pharmacy students had to utilize a drug identification resource to discover that the patient had mixed up his medications. Social work students had an in-depth knowledge of his home living situation and available financial resources, and the medical students listened to breath sounds and reviewed his medical history. Throughout the activity, the participants discover that the patient does not understand his medical condition, medication regimen, and dietary restrictions. Students must identify and share with each other the relevant information about the patient’s medications, living conditions, financial situation, and family support to develop a plan to meet the patient’s medical and nonmedical needs.

Over four hundred students from pharmacy, medicine, social work, and physician assistant programs across the state of Tennessee participated in this activity simultaneously in fall 2020 and fall 2021. The college of pharmacy has three campuses in different cities across the state. The Doctor of Medicine (MD) program participating in the activity was at the same institution while the two social work programs were from another institution. Upperclassmen facilitators recruited from the colleges of pharmacy and medicine led interdisciplinary groups of 10–12 students through the 45-min scenario using a detailed facilitation guide, and faculty representatives from each college facilitated the 10-min prebrief and 30-min debrief discussions.

The same patient case was utilized across both years of the studied intervention. However, the educational approach to implementing this activity differed from the first year to the second. In the first year, students were provided with a prerecorded prebrief video, and then prospectively assigned to small groups with members of each profession. The upperclassmen facilitators were responsible for creating and sending out individual video conferencing links for the day-of the activity, and students joined a separate pre-established Zoom link for the large group debrief after conclusion of their small group discussion. However, the second year, two large Zoom sessions were pre-established, and students joined one of two provided links for a live large-group prebrief. Then, participants were randomly assigned to small groups via the breakout rooms function for the activity. (Although the students were randomized, the breakout room assignments were modified to reflect relatively equal distribution of disciplines across the groups.) In both iterations of the activity, at the end of the small group activity, the students returned to the large group for the final debrief of the exercise.

### 2.2. Research Design and Study Population

This retrospective, matched-pairs study used survey methods over a two-year period (fall 2020 and fall 2021) to evaluate student competency in interprofessional practice before and after a large, virtual IPE activity. The study setting was a health science campus in a large, urban area of the southeastern U.S. This study was reviewed by the Institutional Review Board and determined to be eligible for exempt review under 45 CFR 46.104(d)(1).

The 797 IPE activity participants included second-year student pharmacists (*n* = 336), second-year medical students (*n* = 352), second-year physician assistant students (*n* = 60), and students pursuing a bachelor’s or master’s degree in social work (*n* = 49).

### 2.3. Survey Instrument

All students voluntarily completed an online survey instrument, the Interprofessional Education Collaborative (IPEC) Competency Self-Assessment, Version 3, before and after the IPE activity (Table 1). The IPEC Competency Self-Assessment, Version 3 consists of 16 items that evaluate competency in interprofessional practice and has been used in allied health professions, dentistry, medicine, nursing, pharmacy, social work, and other healthcare education disciplines [2,15]. Each item uses a five-point agreement scale that ranges from strongly disagree (1) to strongly agree (5) and are based on the 42 core competency statements developed by the IPEC expert panel in 2011. The instrument has demonstrated evidence of validity and internal consistency for the two subscales of 0.92 (interprofessional interaction) and 0.96 (interprofessional values) [15].

### 2.4. Data Collection and Analysis

Survey responses were screened for missing data, and incomplete survey responses were excluded from the data analysis. All statistical analyses were performed using IBM SPSS Statistics for Mac, Version 28.0 (IBM Corporation, Armonk, NY, USA). Descriptive statistics were calculated for all variables. As the main intent of the intervention was to impact individuals as opposed to scores, an improved average or median score would not glean any information about the number of individuals who improved because of the IPE activity. Thus, the percentage of individuals showing improvement (positive change) from the pre- to the postsurvey was calculated for each survey item [16]. Likewise, the percentage of individuals showing a decline (negative change) from the pre- to the postsurvey was calculated for each survey item. Wilcoxon signed-rank tests were conducted for all ordinal data to compare pre- and postsurvey results. Subgroup analyses, including Wilcoxon signed-rank tests, were conducted for each discipline. To compare postsurvey results between the two years in which the activity was completed (i.e., 2020 and 2021), a secondary analysis utilizing Mann–Whitney U tests was also performed. All tests were two-tailed with an a priori level of significance of *p* < 0.05.

## 3. Results

A total of 607 students (*n* = 309 in 2020, *n* = 298 in 2021) completed the pre- and postsurveys. Of the participating students, 319 (52.6%) were in pharmacy, 270 (44.5%) in medicine, 9 (1.5%) in social work, 8 (1.3%) in the physician assistant program, and 1 (0.2%) unspecified. The overall survey response rate was 76.2%. Survey response rates were 94.9% for student pharmacists, 76.7% for medical students, 18.4% for social work students, and 13.3% for physician assistant students. Given the low response rates for social work and physician assistant students, subgroup analyses were not conducted for these two.

Results from the surveys from all participants are shown in Table 2. All questions showed statistically significant changes between pre- and postsurveys, demonstrating improved interprofessional competency after participating in the IPE activity.

Results from the surveys from student pharmacist participants are shown in Table 3. All questions showed statistically significant changes between pre- and postsurveys demonstrating improved competency in interprofessional education for student pharmacists after participating in the IPE activity.

Results from the surveys from medical student participants are shown in Table 4. There were statistically significant changes between pre- and postsurveys for all survey items except item 14 (i.e., “I am able to act with honesty and integrity in relationships with other team members”).

A comparison of the results from 2020 versus 2021 for the postsurveys only is shown in Table 5. No significant differences were noted for any of the postsurvey items in 2020 versus 2021.

## 4. Discussion

Overall, implementing a large virtual IPE activity involving a complex patient case and exploring the SODH showed a significant increase in student IPEC-based competencies, regardless of the instructional modality utilized or program discipline analyzed. A significant difference in student IPE competency was also demonstrated in the discipline-specific subgroup analyses for the medicine and pharmacy programs, except for a single competency statement in the medicine cohort. Item 14, “I can act with honesty and integrity in relationships with other team members,” was the only statement in the medicine cohort that failed to show a significant increase in student IPEC competency scoring. However, the initial response data indicated a high level of agreement with this statement (more than 97% indicating agree or strongly agree on the pre-assessment).

As with other studies, our project found that the virtual format alleviated common barriers, including physical space limitations and distractions, geographical constraints, and financial costs [8,17,18]. In this study, no significant difference was noted between the intervention in 2020 and 2021, despite changes in the logistical approach utilized for each activity iteration. Operationally, the modality used in 2020 required a more labor-intensive orientation process for facilitators before the implementation date. Despite more day-of training, the increased time necessary to establish small group breakout rooms, and some technical difficulties with the program, the 2021 methodology resulted in a more streamlined, efficient implementation of the activity while maintaining the same effects on increasing student IPEC competency scores.

Using a virtual format for IPE activities is a promising strategy to facilitate increased participation among a large group of learners, particularly those who may be geographically dispersed [19,20]. Being able to broaden the reach of the IPE activity can lead to increased opportunities to involve health care professions in IPE events that may not otherwise have been involved. However, more direct comparisons of virtual to in-person IPE activities are needed to further investigate whether virtual IPE activities are truly as effective as in-person IPE activities. A 2021 study compared outcomes from an in-person versus a virtual IPE activity and found that the virtual modality was not detrimental to student attitudes and did not adversely affect peer perceptions [17].

This study was unique in that it describes an IPE activity focused on addressing the SDOH. In this activity, participants practiced an authentic team-based approach to provide holistic care for the patient. For example, the case patient had heart failure and struggled with nonmedical concerns such as poor health literacy, lack of transportation, vision issues, and food insecurity. These concerns impacted his desired health outcomes. The medical and nonmedical concerns highlighted accurately that medical providers may not be able to fully address all of these in a patient encounter on their own. However, by working as a team, the participants could see how others in various disciplines could be utilized to provide resources that would result in better patient outcomes for this patient. The participants also discussed and recognized the importance of communication among healthcare disciplines to achieve a common goal. As addressing the SDOH continues to become recognized as an integral component of healthcare, developing educational strategies to teach learners about effective interprofessional coordination to address social risk factors is increasingly important. As this study suggests, education on managing the SDOH may be accomplished in a virtual format. Future research is needed to investigate the application of this knowledge in practice.

Our study has several strengths. First, the study design examined the effect of a virtual IPE patient case across several healthcare-related disciplines using standardized and previously validated IPEC competency statements. Other studies have looked at student perceptions of the activity through questionnaires [21,22,23,24,25,26,27,28,29,30]; however, our survey instruments and the statements generated were derived from the 2011 IPEC framework [18,31]. The virtual design also allowed inclusion of students at distant programs or campuses that may not have been able to participate otherwise. Another strength was the faculty engagement from each of the participating disciplines. The collaboration between medicine, pharmacy, social work, and physician assistant educators to initially create the patient case for this activity was critical in developing a realistic, meaningful, and practice-level appropriate experience. This collaborative approach also helped to ensure that the IPE activity met the competencies of all participating students and their respective fields. Additionally, the creation of a step-by-step facilitator guide for this activity helped to ensure that there were comparable experiences among the small groups.

There were several limitations to our study as well. First, our study population included social work and physician assistant students; however, a poor response rate from these disciplines precluded detection of any changes in IPEC competencies resulting from this activity. Future research must assess the impact on these student populations in this context. Additionally, the virtual activity design, reliance on technology, and video conference platforms were accompanied by several IT-related issues, including difficulty assigning students to small groups and connectivity concerns. Educators should account for this in their planning as it may require adjustments in event timing constraints or alternative accommodation for participants without high-speed Internet access. Furthermore, although this study investigated improvements within the IPEC competencies, the survey tool did not assess whether students’ knowledge of the SDOH improved. Finally, all data analyzed was self-reported and self-assessed by the participating students, which increases the risk response bias. However, standardization to the IPEC competencies helped to minimize this.

Future research may qualitatively investigate the impact of this type of activity. Additionally, as professional education programs recover from the COVID-19 pandemic and return to in-person instruction, future research will explore a virtual versus a hybrid IPE (virtual plus in-person) approach.

## 5. Conclusions

Implementing a large virtual IPE patient case activity exploring SDOH showed a significant increase in student IPEC competencies, regardless of the instructional modality utilized or program discipline. These findings support that a virtual case-based IPE can be a valuable tool for exposing many healthcare students to SDOH. Additionally, these experiences can introduce students to unique factors relevant to patient care and help them understand their roles and responsibilities while working in a healthcare team. Future research is needed to investigate alternative instructional modalities and assess other IPEC competencies not evaluated in this study.

## Figures and Tables

**Table 1:** Items from the Interprofessional Education Collaborative (IPEC) Competency Self-Assessment, Version 3.

Item Number	Survey Statement
1	I am able to choose communication tools and techniques that facilitate effective team interactions.
2	I am able to place the interests of patients at the center of interprofessional health care delivery.
3	I am able to engage other health professionals in shared problem-solving appropriate to the specific care situation.
4	I am able to respect the privacy of patients while maintaining confidentiality in the delivery of team-based care.
5	I am able to inform care decisions by integrating the knowledge and expertise of other professions appropriate to the clinical situation.
6	I am able to embrace the diversity that characterizes the health care team.
7	I am able to apply leadership practices that support effective collaborative practice.
8	I am able to respect the cultures and values of other health professions.
9	I am able to engage other health professionals to constructively manage disagreements about patient care.
10	I am able to develop a trusting relationship with other team members.
11	I am able to use strategies that improve the effectiveness of interprofessional teamwork and team-based care.
12	I am able to demonstrate high standards of ethical conduct in my contributions to team-based care.
13	I am able to use available evidence to inform effective teamwork and team-based practices.
14	I am able to act with honesty and integrity in relationships with other team members.
15	I am able to understand the responsibilities and expertise of other health professions.
16	I am able to maintain competence in my own profession appropriate to my level of training.

**Table 2:** Pre- and postsurvey results demonstrating all students’ self-assessed interprofessional competency (*n* = 607).

Survey Item	Survey	Strongly Disagree, *n* (%)	Disagree, *n* (%)	Neither Agree nor Disagree, *n* (%)	Agree, *n* (%)	Strongly Agree, *n* (%)	Positive Change (%)	Negative Change (%)	*p* Value
Item 1	Pre	2 (0.3%)	7 (1.2%)	48 (7.9%)	380 (62.6%)	170 (28.0%)	40.5%	4.1%	<0.001
	Post	6 (1.0%)	1 (0.2%)	15 (2.5%)	216 (35.6%)	369 (60.8%)			
Item 2	Pre	3 (0.5%)	2 (0.3%)	24 (4.0%)	250 (41.2%)	328 (54.0%)	24.1%	5.3%	<0.001
	Post	4 (0.7%)	0 (0.0%)	9 (1.5%)	155 (25.5%)	439 (72.3%)			
Item 3	Pre	2 (0.3%)	4 (0.7%)	79 (13.0%)	328 (54.0%)	194 (32.0%)	42.8%	4.3%	<0.001
	Post	4 (0.7%)	2 (0.3%)	10 (1.6%)	197 (32.5%)	394 (64.9%)			
Item 4	Pre	2 (0.3%)	1 (0.2%)	36 (5.9%)	233 (38.4%)	335 (55.2%)	21.7%	5.9%	<0.001
	Post	4 (0.7%)	0 (0.0%)	9 (1.5%)	178 (29.3%)	416 (68.5%)			
Item 5	Pre	2 (0.3%)	9 (1.5%)	100 (16.5%)	327 (53.9%)	169 (27.8%)	46.8%	3.3%	<0.001
	Post	4 (0.7%)	0 (0.0%)	13 (2.1%)	205 (33.8%)	385 (63.4%)			
Item 6	Pre	2 (0.3%)	1 (0.2%)	38 (6.3%)	245 (40.4%)	321 (52.9%)	26.2%	5.6%	<0.001
	Post	4 (0.7%)	2 (0.3%)	14 (2.3%)	154 (25.4%)	433 (71.3%)			
Item 7	Pre	2 (0.3%)	6 (1.0%)	71 (11.7%)	317 (52.2%)	211 (34.8%)	35.6%	5.3%	<0.001
	Post	4 (0.7%)	2 (0.3%)	26 (4.3%)	206 (33.9%)	369 (60.8%)			
Item 8	Pre	2 (0.3%)	2 (0.3%)	19 (3.1%)	238 (39.2%)	346 (57.0%)	21.3%	7.4%	<0.001
	Post	4 (0.7%)	1 (0.2%)	11 (1.8%)	169 (27.8%)	422 (69.5%)			
Item 9	Pre	2 (0.3%)	12 (2.0%)	81 (13.3%)	306 (50.4%)	206 (33.9%)	39.7%	4.9%	<0.001
	Post	4 (0.7%)	3 (0.5%)	18 (3.0%)	204 (33.6%)	378 (62.3%)			
Item 10	Pre	2 (0.3%)	0 (0.0%)	41 (6.8%)	314 (51.7%)	250 (41.2%)	32.0%	5.6%	<0.001
	Post	4 (0.7%)	2 (0.3%)	13 (2.1%)	188 (31.0%)	400 (65.9%)			
Item 11	Pre	2 (0.3%)	8 (1.3%)	87 (14.3%)	329 (54.2%)	181 (29.8%)	44.2%	3.5%	<0.001
	Post	4 (0.7%)	3 (0.5%)	22 (3.6%)	188 (31.0%)	390 (64.3%)			
Item 12	Pre	2 (0.3%)	0 (0.0%)	26 (4.3%)	265 (43.7%)	314 (51.7%)	26.2%	5.1%	<0.001
	Post	4 (0.7%)	0 (0.0%)	10 (1.6%)	160 (26.4%)	433 (71.3%)			
Item 13	Pre	3 (0.5%)	4 (0.7%)	55 (9.1%)	303 (49.9%)	242 (39.9%)	34.3%	4.1%	<0.001
	Post	5 (0.8%)	0 (0.0%)	10 (1.6%)	199 (32.8%)	393 (64.7%)			
Item 14	Pre	2 (0.3%)	0 (0.0%)	12 (2.0%)	220 (36.2%)	373 (61.4%)	17.8%	6.1%	<0.001
	Post	4 (0.7%)	0 (0.0%)	8 (1.3%)	154 (25.4%)	441 (72.7%)			
Item 15	Pre	2 (0.3%)	12 (2.0%)	63 (10.4%)	309 (50.9%)	221 (36.4%)	36.4%	5.3%	<0.001
	Post	5 (0.8%)	4 (0.7%)	14 (2.3%)	204 (33.6%)	380 (62.6%)			
Item 16	Pre	2 (0.3%)	1 (0.2%)	42 (6.9%)	298 (49.1%)	264 (43.5%)	30.0%	3.3%	<0.001
	Post	4 (0.7%)	0 (0.0%)	13 (2.1%)	185 (30.5%)	405 (66.7%)			

**Table 3:** Pre- and postsurvey results demonstrating student pharmacists’ self-assessed interprofessional competency (*n* = 319).

Survey Item	Survey	Strongly Disagree, *n* (%)	Disagree, *n* (%)	Neither Agree nor Disagree, *n* (%)	Agree, *n* (%)	Strongly Agree, *n* (%)	Positive Change (%)	Negative Change (%)	*p* Value
Item 1	Pre	1 (0.3%)	6 (1.9%)	18 (5.6%)	202 (63.3%)	92 (28.8%)	47.3%	2.5%	<0.001
	Post	2 (0.6%)	0 (0.0%)	6 (1.9%)	86 (27.0%)	225 (70.5%)			
Item 2	Pre	2 (0.6%)	2 (0.6%)	10 (3.1%)	123 (38.6%)	182 (57.1%)	25.7%	3.4%	<0.001
	Post	2 (0.6%)	0 (0.0%)	3 (0.9%)	62 (19.4%)	252 (79.0%)			
Item 3	Pre	1 (0.3%)	2 (0.6%)	37 (11.6%)	175 (54.9%)	104 (32.6%)	47.6%	2.8%	<0.001
	Post	2 (0.6%)	0 (0.0%)	3 (0.9%)	82 (25.7%)	232 (72.7%)			
Item 4	Pre	1 (0.3%)	1 (0.3%)	14 (4.4%)	113 (35.4%)	190 (59.6%)	22.3%	6.3%	<0.001
	Post	2 (0.6%)	0 (0.0%)	4 (1.3%)	77 (24.1%)	236 (74.0%)			
Item 5	Pre	1 (0.3%)	6 (1.9%)	39 (12.2%)	168 (52.7%)	105 (32.9%)	45.1%	2.8%	<0.001
	Post	2 (0.6%)	0 (0.0%)	3 (0.9%)	88 (27.6%)	226 (70.8%)			
Item 6	Pre	1 (0.3%)	1 (0.3%)	15 (4.7%)	133 (41.7%)	169 (53.0%)	29.5%	4.7%	<0.001
	Post	2 (0.6%)	0 (0.0%)	4 (1.3%)	70 (21.9%)	243 (76.2%)			
Item 7	Pre	1 (0.3%)	4 (1.3%)	31 (9.7%)	159 (49.8%)	124 (38.9%)	38.6%	4.7%	<0.001
	Post	2 (0.6%)	1 (0.3%)	10 (3.1%)	80 (25.1%)	226 (70.8%)			
Item 8	Pre	1 (0.3%)	2 (0.6%)	6 (1.9%)	119 (37.3%)	191 (59.9%)	23.8%	6.0%	<0.001
	Post	2 (0.6%)	0 (0.0%)	2 (0.6%)	71 (22.3%)	244 (76.5%)			
Item 9	Pre	1 (0.3%)	6 (1.9%)	39 (12.2%)	157 (49.2%)	116 (36.4%)	43.9%	4.1%	<0.001
	Post	2 (0.6%)	0 (0.0%)	5 (1.6%)	88 (27.6%)	224 (70.2%)			
Item 10	Pre	1 (0.3%)	0 (0.0%)	25 (7.8%)	151 (47.3%)	142 (44.5%)	36.1%	3.4%	<0.001
	Post	2 (0.6%)	0 (0.0%)	3 (0.9%)	78 (24.5%)	236 (74.0%)			
Item 11	Pre	1 (0.3%)	3 (0.9%)	40 (12.5%)	169 (53.0%)	106 (33.2%)	48.0%	1.9%	<0.001
	Post	2 (0.6%)	1 (0.3%)	4 (1.3%)	74 (23.2%)	238 (74.6%)			
Item 12	Pre	1 (0.3%)	0 (0.0%)	10 (3.1%)	146 (45.8%)	162 (50.8%)	31.3%	4.4%	<0.001
	Post	2 (0.6%)	0 (0.0%)	4 (1.3%)	66 (20.7%)	247 (77.4%)			
Item 13	Pre	1 (0.3%)	3 (0.9%)	20 (6.3%)	159 (49.8%)	136 (42.6%)	36.4%	2.5%	<0.001
	Post	2 (0.6%)	0 (0.0%)	3 (0.9%)	79 (24.8%)	235 (73.7%)			
Item 14	Pre	1 (0.3%)	0 (0.0%)	5 (1.6%)	123 (38.6%)	190 (59.6%)	23.8%	3.1%	<0.001
	Post	2 (0.6%)	0 (0.0%)	2 (0.6%)	60 (18.8%)	255 (79.9%)			
Item 15	Pre	1 (0.3%)	3 (0.9%)	25 (7.8%)	171 (53.6%)	119 (37.3%)	40.8%	3.4%	<0.001
	Post	2 (0.6%)	0 (0.0%)	4 (1.3%)	81 (25.4%)	232 (72.7%)			
Item 16	Pre	1 (0.3%)	0 (0.0%)	22 (6.9%)	153 (48.0%)	143 (44.8%)	35.7%	2.2%	<0.001
	Post	2 (0.6%)	0 (0.0%)	3 (0.9%)	76 (23.8%)	238 (74.6%)			

**Table 4:** Pre- and postsurvey results demonstrating medical students’ self-assessed interprofessional competency (*n* = 270).

Survey Item	Survey	Strongly Disagree, *n* (%)	Disagree, *n* (%)	Neither Agree nor Disagree, *n* (%)	Agree, *n* (%)	Strongly Agree, *n* (%)	Positive Change (%)	Negative Change (%)	*p* Value
Item 1	Pre	1 (0.4%)	1 (0.4%)	28 (10.4%)	167 (61.9%)	73 (27.0%)	32.6%	5.2%	<0.001
	Post	3 (1.1%)	1 (0.4%)	7 (2.6%)	125 (46.3%)	134 (49.6%)			
Item 2	Pre	1 (0.4%)	0 (0.0%)	12 (4.4%)	119 (44.1%)	138 (51.1%)	21.5%	7.4%	<0.001
	Post	2 (0.7%)	0 (0.0%)	5 (1.9%)	89 (33.0%)	174 (64.4%)			
Item 3	Pre	1 (0.4%)	1 (0.4%)	37 (13.7%)	144 (53.3%)	87 (32.2%)	36.7%	5.6%	<0.001
	Post	2 (0.7%)	1 (0.4%)	6 (2.2%)	110 (40.7%)	151 (55.9%)			
Item 4	Pre	1 (0.4%)	0 (0.0%)	18 (6.7%)	114 (42.2%)	137 (50.7%)	21.1%	5.9%	<0.001
	Post	2 (0.7%)	0 (0.0%)	4 (1.5%)	96 (35.6%)	168 (62.2%)			
Item 5	Pre	1 (0.4%)	3 (1.1%)	55 (20.4%)	150 (55.6%)	61 (22.6%)	47.8%	4.1%	<0.001
	Post	2 (0.7%)	0 (0.0%)	9 (3.3%)	112 (41.5%)	147 (54.4%)			
Item 6	Pre	1 (0.4%)	0 (0.0%)	21 (7.8%)	105 (38.9%)	143 (53.0%)	22.6%	7.0%	<0.001
	Post	2 (0.7%)	2 (0.7%)	9 (3.3%)	79 (29.3%)	178 (65.9%)			
Item 7	Pre	1 (0.4%)	2 (0.7%)	37 (13.7%)	146 (54.1%)	84 (31.1%)	31.1%	6.3%	<0.001
	Post	2 (0.7%)	1 (0.4%)	15 (5.6%)	120 (44.4%)	132 (48.9%)			
Item 8	Pre	1 (0.4%)	0 (0.0%)	12 (4.4%)	111 (41.1%)	146 (54.1%)	18.5%	9.3%	0.044
	Post	2 (0.7%)	1 (0.4%)	7 (2.6%)	94 (34.8%)	166 (61.5%)			
Item 9	Pre	1 (0.4%)	6 (2.2%)	38 (14.1%)	139 (51.5%)	86 (31.9%)	34.4%	5.9%	<0.001
	Post	2 (0.7%)	2 (0.7%)	12 (4.4%)	111 (41.1%)	143 (53.0%)			
Item 10	Pre	1 (0.4%)	0 (0.0%)	15 (5.6%)	151 (55.9%)	103 (38.1%)	28.1%	7.0%	<0.001
	Post	2 (0.7%)	1 (0.4%)	8 (3.0%)	104 (38.5%)	155 (57.4%)			
Item 11	Pre	1 (0.4%)	4 (1.5%)	44 (16.3%)	150 (55.6%)	71 (26.3%)	39.6%	4.8%	<0.001
	Post	2 (0.7%)	1 (0.4%)	17 (6.3%)	108 (40.0%)	142 (52.6%)			
Item 12	Pre	1 (0.4%)	0 (0.0%)	14 (5.2%)	112 (41.5%)	143 (53.0%)	20.4%	5.6%	<0.001
	Post	2 (0.7%)	0 (0.0%)	5 (1.9%)	88 (32.6%)	175 (64.8%)			
Item 13	Pre	2 (0.7%)	1 (0.4%)	32 (11.9%)	137 (50.7%)	98 (36.3%)	32.2%	5.9%	<0.001
	Post	3 (1.1%)	0 (0.0%)	6 (2.2%)	114 (42.2%)	147 (54.4%)			
Item 14	Pre	1 (0.4%)	0 (0.0%)	6 (2.2%)	91 (33.7%)	172 (63.7%)	11.1%	9.3%	0.800
	Post	2 (0.7%)	0 (0.0%)	5 (1.9%)	88 (32.6%)	175 (64.8%)			
Item 15	Pre	1 (0.4%)	8 (3.0%)	36 (13.3%)	129 (47.8%)	96 (35.6%)	30.7%	7.4%	<0.001
	Post	3 (1.1%)	4 (1.5%)	8 (3.0%)	119 (44.1%)	136 (50.4%)			
Item 16	Pre	1 (0.4%)	1 (0.4%)	18 (6.7%)	138 (51.1%)	112 (41.5%)	23.3%	4.8%	<0.001
	Post	2 (0.7%)	0 (0.0%)	9 (3.3%)	105 (38.9%)	154 (57.0%)			

**Table 5:** Postsurvey results comparing students’ self-assessed interprofessional competency in 2020 versus 2021 (*n* = 607).

Survey Item	Survey Year	Strongly Disagree, *n* (%)	Disagree, *n* (%)	Neither Agree nor Disagree, *n* (%)	Agree, *n* (%)	Strongly Agree, *n* (%)	*p* Value
Item 1	2020	2 (0.6%)	0 (0.0%)	8 (2.6%)	116 (37.5%)	183 (59.2%)	0.495
	2021	4 (1.3%)	1 (0.3%)	7 (2.3%)	100 (33.6%)	186 (62.4%)	
Item 2	2020	2 (0.6%)	0 (0.0%)	4 (1.3%)	85 (27.5%)	218 (70.6%)	0.349
	2021	2 (0.7%)	0 (0.0%)	5 (1.7%)	70 (23.5%)	221 (74.2%)	
Item 3	2020	2 (0.6%)	1 (0.3%)	5 (1.6%)	104 (33.7%)	197 (63.8%)	0.563
	2021	2 (0.7%)	1 (0.3%)	5 (1.7%)	93 (31.2%)	197 (66.1%)	
Item 4	2020	2 (0.6%)	0 (0.0%)	3 (1.0%)	94 (30.4%)	210 (68.0%)	0.826
	2021	2 (0.7%)	0 (0.0%)	6 (2.0%)	84 (28.2%)	206 (69.1%)	
Item 5	2020	2 (0.6%)	0 (0.0%)	5 (1.6%)	106 (34.3%)	196 (63.4%)	0.921
	2021	2 (0.7%)	0 (0.0%)	8 (2.7%)	99 (33.2%)	189 (63.4%)	
Item 6	2020	2 (0.6%)	0 (0.0%)	7 (2.3%)	80 (25.9%)	220 (71.2%)	0.991
	2021	2 (0.7%)	2 (0.7%)	7 (2.3%)	74 (24.8%)	213 (71.5%)	
Item 7	2020	2 (0.6%)	0 (0.0%)	13 (4.2%)	112 (36.2%)	182 (58.9%)	0.414
	2021	2 (0.7%)	2 (0.7%)	13 (4.4%)	94 (31.5%)	187 (62.8%)	
Item 8	2020	2 (0.6%)	0 (0.0%)	5 (1.6%)	91 (29.4%)	211 (68.3%)	0.557
	2021	2 (0.7%)	1 (0.3%)	6 (2.0%)	78 (26.2%)	211 (70.8%)	
Item 9	2020	2 (0.6%)	1 (0.3%)	11 (3.6%)	103 (33.3%)	192 (62.1%)	0.888
	2021	2 (0.7%)	2 (0.7%)	7 (2.3%)	101 (33.9%)	186 (62.4%)	
Item 10	2020	2 (0.6%)	1 (0.3%)	5 (1.6%)	101 (32.7%)	200 (64.7%)	0.616
	2021	2 (0.7%)	1 (0.3%)	8 (2.7%)	87 (29.2%)	200 (67.1%)	
Item 11	2020	2 (0.6%)	1 (0.3%)	12 (3.9%)	102 (33.0%)	192 (62.1%)	0.294
	2021	2 (0.7%)	2 (0.7%)	10 (3.4%)	86 (28.9%)	198 (66.4%)	
Item 12	2020	2 (0.6%)	0 (0.0%)	4 (1.3%)	84 (27.2%)	219 (70.9%)	0.847
	2021	2 (0.7%)	0 (0.0%)	6 (2.0%)	76 (25.5%)	214 (71.8%)	
Item 13	2020	2 (0.6%)	0 (0.0%)	3 (1.0%)	109 (35.3%)	195 (63.1%)	0.505
	2021	3 (1.0%)	0 (0.0%)	7 (2.3%)	90 (30.2%)	198 (66.4%)	
Item 14	2020	2 (0.6%)	0 (0.0%)	2 (0.6%)	80 (25.9%)	225 (72.8%)	0.850
	2021	2 (0.7%)	0 (0.0%)	6 (2.0%)	74 (24.8%)	216 (72.5%)	
Item 15	2020	2 (0.6%)	2 (0.6%)	7 (2.3%)	113 (36.6%)	185 (59.9%)	0.194
	2021	3 (1.0%)	2 (0.7%)	7 (2.3%)	91 (30.5%)	195 (65.4%)	
Item 16	2020	2 (0.6%)	0 (0.0%)	4 (1.3%)	102 (33.0%)	201 (65.0%)	0.484
	2021	2 (0.7%)	0 (0.0%)	9 (3.0%)	83 (27.9%)	204 (68.5%)	

## Data Availability

Data are not available for sharing due to ethical, legal, and privacy restrictions.
